# Morphological Outer Retina Findings in Multiple Sclerosis Patients With or Without Optic Neuritis

**DOI:** 10.3389/fneur.2020.00858

**Published:** 2020-09-15

**Authors:** Lucia Ziccardi, Lucilla Barbano, Laura Boffa, Maria Albanese, Andrzej Grzybowski, Diego Centonze, Vincenzo Parisi

**Affiliations:** ^1^Istituto di Ricovero e Cura a Carattere Scientifico - Fondazione Bietti, Rome, Italy; ^2^Unit of Neurology, Department of Systems Medicine, Tor Vergata University, Rome, Italy; ^3^Department of Ophthalmology, University of Warmia and Mazury, Olsztyn, Poland; ^4^Institute for Research in Ophthalmology, Foundation for Ophthalmology Development, Poznan, Poland; ^5^Istituto di Ricovero e Cura a Carattere Scientifico Neuromed - Unit of Neurology and Neurorehabilitation, Pozzilli, Italy

**Keywords:** multiple sclerosis, optic neuritis (ON), SD-OCT imaging, neurodegeneration, outer retina

## Abstract

**Purpose:** To investigate on the morphology of the macular inner (IR) and outer (OR) layers in multiple sclerosis (MS) patients with and without history of optic neuritis (ON), followed by good or poor recovery of best corrected visual acuity (BCVA).

**Methods:** Thirty-five normal control subjects and 93 relapsing remitting MS patients were enrolled. Of this, 40 MS patients without ON (MS-noON, 40 eyes), 27 with history of ON and good BCVA recovery (MS-ON-G, 27 eyes), and 26 with history of ON and poor BCVA recovery (MS-ON-P, 26 eyes) were studied. Controls and MS patients underwent an extensive ophthalmological examination including spectral-domain optical coherence tomography evaluating in 3 localized macular areas (0–1 mm, Area 1; 1–3 mm, Area 2; 3- 6 mm, Area 3), volumes (MV), and thicknesses (MT) of the whole retina (WR), further segmented in IR and OR. The differences of MV and MT between the groups were tested by ANOVA. In the MS-ON-P group, the correlations between MV and MT and BCVA were evaluated by Pearson's test.

**Results:** When compared to controls, the MS-noON group showed not significantly (*p* > 0.01) different MVs, whereas MTs were significantly (*p* < 0.01) reduced in the evaluation of WR and IR. In the MS-ON-G group, a significant (*p* < 0.01) reduction of WR and IR MVs and MTs was found in Areas 2 and 3; OR MVs and MTs were similar (*p* > 0.01) to controls. In the MS-ON-P group a significant (*p* < 0.01) reduction of WR, IR, and OR MVs and MTs was detected in all areas; the BCVA reduction was significantly (*p* < 0.01) correlated with WR and IR MVs and MTs.

**Conclusions:** In MS without history of ON or when ON is followed by a good BCVA recovery, the neurodegenerative process is limited to IR macular layers; in the presence of ON, with a poor BCVA recovery, a morphological impairment of both IR and OR macular layers occurs.

## Introduction

In about 20–25% of multiple sclerosis (MS) patients, the pathology can onset with retrobulbar optic neuritis (ON) (1), that is followed by a secondary neurodegenerative process, reflecting retrograde degeneration that involves retinal ganglion cells (RGCs) and their axons (2).

The effects of this neurodegenerative process on the neuro-retinal structure can be objectively studied by Optical Coherence Tomography (OCT), that usually detects a reduction of retinal nerve fibers layer (RNFL) thickness (3–5).

An interesting and widely discussed topic in MS with or without ON has been to investigate whether the retinal elements of the macular region are morphologically impaired.

Currently, with the innovative Spectral domain-OCT (Sd-OCT) technique, it is possible to selectively segment the macular volume (MV) and thickness (MT) of the outer (OR) and inner retinal (IR) layers (6). IR abnormalities [thinning of RGCs/inner plexiform layer (GC/IPL) and thinning/thickening of the inner nuclear layer (INL) and reduced whole macular volume (4, 7–10)] are known to occur in MS (11–13) with and without previous optic neuritis, whereas data about morphological macular OR changes are controversial (7, 12, 14–19). All this suggests that the MS neurodegenerative process involves the macular layers and mainly the ganglionic elements located in the IR.

However, it is not yet entirely clarified whether the neurodegeneration could extend beyond the level of the INL (14) toward the macular pre-ganglionic elements, thus involving photoreceptors and bipolar cells forming the OR (20). Indeed, it is of great interest to identify whether there is a morphological involvement of specific IR and/or OR macular elements in the neurodegenerative process of MS, in the occurrence of ON or not, and whether the macular morphological condition is linked to the recovery of visual acuity after the ON event.

Therefore, our aim was to evaluate the morphology of IR and OR layers in localized macular areas in MS patients with and without history of ON, with good or poor recovery of high-contrast best corrected visual acuity (BCVA). Relative results obtained in MS patients might identify whether macular OR layers are morphologically involved in the MS neurodegenerative process, contributing to this widely debated topic. In addition, we aimed to assess whether the OR and IR morphology may be related to the good or poor recovery of BCVA or not.

## Materials and Methods

### Participants

All research procedures described in this work adhered to the tenets of the Declaration of Helsinki. The study protocol was approved by the local Ethical Committee (Comitato Etico Centrale IRCCS Lazio, Sezione IFO/Fondazione Bietti, Rome, Italy) and upon recruitment, informed consent after a full explanation of the procedure was obtained from each subject enrolled in the study.

Ninety-three relapsing remitting (RR) MS patients were enrolled at the Visual Neurophysiology and Neuro-Ophthalmology Research Unit, IRCCS- Fondazione Bietti referred by the Neurology Department of Tor Vergata Policlinic of Rome, between January 2014 and September 2018.

In order to obtain homogeneous MS groups (with ON and without ON followed by poor or good recovery of VA, see below), the MS patients were selected form a large cohort (*n* = 358) based on the following demographic and clinical characteristics:

1. Age between 28 and 45 years;
2. Diagnosis of RR MS according to validated 2010 McDonald criteria (21);
3. MS disease duration (MS-DD), estimated as the number of years from onset to the most recent assessment of disability, ranging from 5 to 22 years;
4. Expanded Disability Status Scale (EDSS), as ten-point disease severity derived from nine ratings for individual neurological domains (22), ranging from 0 to 3; this score was assessed by two trained (Neurostatus: available at http://www.neurostatus.net/index.php?file=start) neurologists (LaB and MA)
5. Treatment with disease-modifying therapies (DMT) currently approved for preventing MS relapses. DMT considered in our study were Interferon-β-1a, Interferon-β-1b, Peginterferon beta-1a, Glatiramer acetate, Natalizumab, Dimethyl fumarate, and Teriflunomide (23).
6. Absence of ON or a single episode of ON without recurrence, that elapsed from the onset of the disease at least 12 months (range 13–20 months) before the inclusion in the study. For MS patients with ON, this criteria was chosen, since it is known that the retrograde degeneration following ON occurs over a period of 6 months (24). When a MS patient was affected by ON in both eyes, we studied the eye affected longer that met the inclusion criteria.
7. Based on the ophthalmological examination, other inclusion criteria were: absence of glaucoma or other diseases involving cornea, lens, uvea, and retina; absence of systemic diseases (i.e., diabetes); BCVA between 0 and 1 LogMAR of the Early Treatment of Diabetic Retinopathy (ETDRS) charts; absence of central visual field defects and ability to maintain a stable fixation that allowed a Sd-OCT scan to be performed (see below).

A group of selected 35 age-matched healthy subjects (mean age: 39.5 ± 5.4 years), providing 35 normal eyes, with BCVA of 0.0 LogMAR, served as controls.

The selected MS patients were divided into two groups on the basis of previous history of ON or not. In the assignment to the two groups, similar age, MS-DD, and EDSS values were considered.

A total of 40 MS patients (mean age 40.6 ± 3.9 years; 26 females and 14 males; mean MS-DD 8.6 ± 4.23 years, range 5–21 years; mean EDSS score 1.48 ± 1.10, range 0–3) without history of unilateral or bilateral clinical signs of ON (i.e., painless reduction of BCVA, contrast sensitivity, color vision, and any type of visual filed defects) and a high-contrast BCVA of 0.0 logMAR were included. When both eyes met the inclusion criteria, only one eye was randomly chosen for the study. Therefore, we considered 40 eyes from 40 MS patients without ON (MS-noON Group).

A total of 53 MS patients (mean age 38.9 ± 4.2 years; 32 females and 21 males) with previous history of unilateral or bilateral ON were included. They were further divided in to two groups on the basis of the recovery of BCVA after ON:

A total of 27 MS patients (mean age 38.2 ± 4.7 years; 17 females and 10 males; mean MS-DD 9.2 ± 6.2 years, range 5–22 years; mean EDSS score 1.59 ± 1.02, range 0–3) with previous history of single unilateral or bilateral ON and with “good” recovery of high-contrast BCVA (0.0 logMAR) after ON were included. Therefore, we considered 27 eyes from 27 MS patients with ON for the (MS-ON-G) group;

A total of 26 MS patients (mean age 39.4 ± 3.8 years; 15 females and 11 males; mean MS-DD 9.4 ± 6.6 years, range 5–22 years; mean EDSS score 1.62 ± 1.08, range 0–3) with previous history of single unilateral or bilateral ON with “poor” recovery of high-contrast BCVA (between 0.2 and 1 logMAR) after ON were chosen. Therefore, we considered 26 eyes from 26 MS patients with ON for the (MS-ON-P) group.

Based on the previous mentioned inclusion criteria, the MS groups with or without ON were homogeneous for age, MS-DD, and EDSS and the MS groups with ON were homogeneous for the number of ON and for the time elapsed from ON (see below section Demographic and Clinical Features and Table 1).

**Table 1:** Demographic and clinical features in Multiple Sclerosis patients without Optic Neuritis (MS-noON), with Optic Neuritis and good recovery of best corrected visual acuity (MS-ON-G) and with Optic Neuritis and poor recovery of best corrected visual acuity (MS-ON-P).

	**MS-noON** **(*N* = 40)** **mean ± 1SD**	**MS-ON-G** **(*N* = 27)** **mean ± 1SD**	**MS-ON-P** **(*N* = 26)** **mean ± 1SD**
**Age (years)**	40.6 ± 3.9	38.2 ± 4.7^§^	39.4 ± 3.8^§^^,^ ^#^
**MS-DD (years)**	8.6 ± 4.23	9.2 ± 6.2^§^	9.4 ± 6.6^§^^,^ ^#^
**EDSS score**	1.5 ± 1.1	1.6 ± 1.0^§^	1.6 ± 1.1^§^^,^ ^#^
**ON (N)**	-	1.0 ± 0.0	1.0 ± 0.0^#^
**Time elapsed from ON (months)**	-	15.3 ± 2.4	14.9 ± 2.7^#^

N, number; SD, one Standard Deviation of the mean; MS-DD, Multiple Sclerosis Disease Duration; EDSS, Expanded Disability Status Scale; ON, optic neuritis. One-way analysis of variance between groups:

§ *p > 0.01 vs. MS-noON group*,

# *p > 0.01 vs. MS-ON-G group*.

### Sd-OCT Assessment

The retinal morphology can be explored *in vivo* by Sd-OCT, providing layer-by-layer objective measurements of anatomical structures related to the macular area (25). Sd-OCT scans were obtained in a dark room after pupil dilation with tropicamide 1% eye drops and each scan was carefully reviewed for the accurate identification and segmentation of the retinal layers by two expert graders (LZ, LuB) to exclude cases of failed segmentation. Quality control and APOSTEL recommendations according to the published criteria were followed (26, 27). The OCT image quality signal strength index of the acquired scan was at least 40. Scans that did not fulfill the above criteria were excluded from the analysis.

We used the RTVue-100 device version 6.3 (Optovue, Fremont, CA), which uses a low-coherence light source centered at 840 nm with 50 nm bandwidth, which gives an axial resolution of 5 micrometers.

By using the MM5 protocol, we collected MV and MT data from the ETDRS 9 regions map. The MM5 grid scanning protocol consists of 11 horizontal lines with 5 mm scan length, 6 horizontal lines with 3 mm scan length, 11 vertical lines with 5 mm scan length, and 6 vertical lines with 3 mm scan length each at 0.5 mm intervals, all centered at the fovea. The number of A-scans in long horizontal and vertical lines is 668 and the number of A-scans in short horizontal and vertical lines is 400. This scan configuration provided an acquisition rate of 26.000 A-scans /second.

The segmentation algorithm of the MM5 scanning protocol also enables the automatic segmentation of MV and MT, of whole retina (WR), IR, and OR from the square grid centered on fixation target. The software automatically divides the inner and outer neurosensory retinas at the boundary between the INL and the outer plexiform layer (OPL). The OR encloses the OPL, the outer nuclear layer, and the photoreceptor layer. The IR examines the RNFL, the GC/IPL, and the INL. The boundaries of the OR were the posterior of the OPL and the photoreceptor inner segment/outer segment junction. The following boundaries were identified for the IR segmentation: the inner limiting membrane and the posterior of the INL.

Retinal thickness was generated automatically as thickness is measured between the two interfaces (the vitreoretinal surface and the basement membrane of the RPE-Bruch membrane complex) at each measurement point along the scan's x-axis. We selected the MT map analysis protocol on the device to display the numeric averages of the measurements in each of the 9 ETDRS map sectors. A 3D model of the retina was computed and MV were assessed for each of the subfields (within 1, 3, and 6 mm, respectively) as defined by the ETDRS. Mean values of MV and MT from a circular 1 mm area and from annular ETDRS regions outside the 1 mm central one were calculated averaging the supero-infero-nasal-temporal values.

For OR, IR, and for the whole retina (WR=IR+OR), the software provides mean volumes and thicknesses (within 1, 3, and 6 mm, measured in mm3 and microns, respectively) that are displayed topographically in each of the 9 ETDRS map sectors.

We considered MVs of WR, IR, and OR measured within:

1. the 1 mm central area (named as Area 1, directly provided by the Sd-OCT machine)
2. the middle 1–3 mm ring (named as Area 2, obtained by subtracting from the displayed volume within 3 mm of the ones within the 1 mm area),
3. the external 3–6 mm ring (named as Area 3, obtained by subtracting from the displayed volume within 6 mm of the one within 3 mm directly provided by the Sd-OCT machine),
4. the whole 6 mm area (named as Area 1+Area 2+ Area 3, directly provided by the Sd-OCT machine).

We also analyzed MTs of WR, IR, and OR from Areas 1, 2, and 3 as provided directly from the device for the ETDRS map: foveal (0–1 mm), perifoveal (1–3 mm), and parafoveal (3–6 mm) areas, respectively.

Mean values of MV and MT from circular/annular ETDRS regions were calculated averaging the supero-infero-nasal-temporal values.

### Statistical Analysis

The differences of age, MS-DD, and EDSS between the MS-noON, MS-ON-G, and MS-ON-P groups were evaluated by the one-way analysis of variance (ANOVA). The differences of the number of ON and the time elapsed from the ON between the MS-ON-G and MS-ON- P groups were evaluated by the ANOVA.

The difference of mean values of Sd-OCT parameters (MVs and MTs) detected in the controls, MS-noON, MS-ON-G, and MS-ON-P groups were also evaluated by ANOVA. A *p*-value of 0.01 was chosen as significant to compensate for multiple comparisons. Moreover, in the MS-ON-P group, multiple regression analysis was performed between BCVA and WR, IR, and OR MV and MT values, respectively. As usual, we chose a *p*-value of 0.05 as significant. Minitab 17 (version 1) software was used for statistical analysis.

## Results

### Demographic and Clinical Features

On Table 1 we reported the demographic and clinical features observed in the MS-noON, MS-ON-G, and MS-ON-P groups. The descriptive statistics of age, MS-DD, and EDSS values were not significantly different between the MS-noON, MS-ON-G, and MS-ON-P groups. The descriptive statistics of the number of ON and the time elapsed from ON were not significantly different between the MS-ON-G and MS-ON-P groups.

### Sd-OCT Macular Volume Data

**Figure 1:**
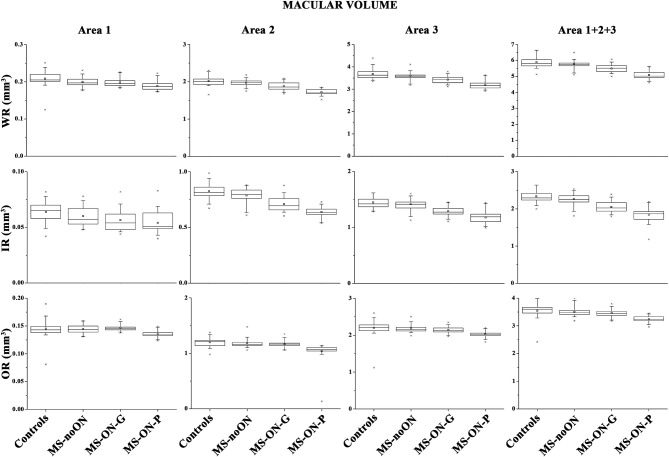
Box plots of Spectral domain-Optical Coherence Tomography macular volumes in control (C) eyes, Multiple Sclerosis patients without Optic Neuritis (MS-noON), with Optic Neuritis and good recovery of best corrected visual acuity (MS-ON-G), and with Optic Neuritis and poor recovery of best corrected visual acuity (MS-ON-P). WR-MV= Whole Retinal Macular Volume; IR-MV= Inner Area Macular Volume; OR-MV = Outer Retina Macular Volume; Area 1= circular area 1 mm centered to the fovea; Area 2 = annular area 1–3 mm centered to the fovea; Area 3 = annular area 3–6 mm centered to the fovea; Area 1+2+3 = whole area within 6 mm.

On Figure 1 we presented the box plots of the values of the WR, IR, and OR MV measured from each localized area observed in the control, MS-noON, MS-ON-G, and MS-ON-P groups. On Table 2, the statistical analysis between groups is also reported.

**Table 2:** Spectral domain-Optical Coherence Tomography macular volume (MV) segmentation analysis.

		**WR-MV**				**IR-MV**				**OR-MV**			
		**AREA 1**	**AREA 2**	**AREA 3**	**AREA 1+2+3**	**AREA 1**	**AREA 2**	**AREA 3**	**AREA 1+2+3**	**AREA 1**	**AREA 2**	**AREA 3**	**AREA 1+2+3**
**MS-noON** **vs. C**	*f* (1,74)	3.265	2.441	3.321	3.522	0.602	4.299	1.152	2.563	0.132	1.078	0.299	0.611
	*P*	0.075	0.123	0.073	0.048	0.439	0.042	0.228	0.114	0.729	0.302	0.558	0.436
**MS-ON-G** **vs. C**	*f* (1,61)	4.011	16.429	21.632	25.858	1.789	32.222	28.035	35.792	0.111	2.862	1.614	2.295
	*P*	0.050	** <0.01**	** <0.01**	** <0.01**	0.186	** <0.01**	** <0.01**	** <0.01**	0.756	0.096	0.210	0.135
**MS-ON-G** **vs. MS-noON**	*f* (1,66)	0.132	14.301	15.789	17.041	0.632	23.222	27.403	19.859	1.642	0.789	2.588	1.783
	*P*	0.716	** <0.01**	** <0.01**	** <0.01**	0.431	** <0.01**	** <0.01**	** <0.01**	0.205	0.337	0.112	0.186
**MS-ON-P** **vs. C**	*f* (1,60)	41.22	99.75	70.66	89.02	0.22	119.75	68.36	83.40	11.09	19.24	13.71	25.54
	*P*	** <0.01**	** <0.01**	** <0.01**	** <0.01**	0.639	** <0.01**	** <0.01**	** <0.01**	** <0.01**	** <0.01**	** <0.01**	** <0.01**
**MS-ON-P** **vs. MS-noON**	*f* (1,65)	19.25	154.88	78.47	106.01	4.47	97.52	76.88	85.02	35.88	18.23	41.86	52.96
	*P*	** <0.01**	** <0.01**	** <0.01**	** <0.01**	0.038	** <0.01**	** <0.01**	** <0.01**	** <0.01**	** <0.01**	** <0.01**	** <0.01**
**MS-ON-P** **vs. MS-ON-G**	*f* (1,52)	14.51	42.78	23.35	32.08	0.97	20.75	14.66	16.71	62.76	10.56	23.17	36.36
	*P*	** <0.01**	** <0.01**	** <0.01**	** <0.01**	0.328	** <0.01**	** <0.01**	** <0.01**	** <0.01**	** <0.01**	** <0.01**	** <0.01**

*Results of statistical analysis (one-way analysis of variance) between groups: control (C) eyes, Multiple Sclerosis patients without Optic Neuritis (MS-noON), with Optic Neuritis and good recovery of best corrected visual acuity (MS-ON-G), and with Optic Neuritis and poor recovery of best corrected visual acuity (MS-ON-P). WR-MV, Whole Retinal Macular Volume; IR-MV, Inner Area Macular Volume; OR-MV, Outer Retina Macular Volume; Area 1 = circular area 1 mm centered to the fovea; Area 2 = annular area 1–3 mm centered to the fovea; Area 3 = annular area 3–6 mm centered to the fovea; Area 1+2+3 = whole area within 6 mm; p < 0.01 were considered as statistically significant for group comparisons and are in bold*.

On average, in the MS-noON group, the values of WR, IR, and OR MV detected in Areas 1, 2, and 3 and in the combined Area 1+2+3 were not significantly (*p* > 0.01) reduced with respect to controls.

In both the MS-ON-G and MS-ON-P groups, mean values of WR and IR MV detected in localized Areas 2 and 3 as well as in the combined Area 1+ Area 2 + Area 3 were significantly (*p* < 0.01) reduced when compared to controls and to MS-noON group. In the same areas, the mean values of WR and IR MV observed in the MS-ON-P group were further significantly (*p* < 0.01) reduced with respect to those detected in the MS-ON-G group. The values of WR MV detected in Area 1 were significantly (*p* < 0.01) reduced in the MS-ON-P group with respect to those of the controls, MS-noON, and MS-ON-G groups, whereas, in the same Area 1, IR MV values were not significantly reduced (*p* > 0.01).

When considering the OR MVs, not significant (*p* > 0.01) differences between the values observed in the controls, MS-noON, and MS-ON-G groups in any Area (1, 2, 3, or 1+2+3) were found. On the contrary, the values of OR MVs detected in all Areas (1, 2, 3, or 1+2+3) in the MS-ON-P group were significantly (*p* < 0.01) reduced with respect to those of the controls, MS-noON, and MS-ON-G groups.

### Sd-OCT Macular Thickness Data

**Figure 2:**
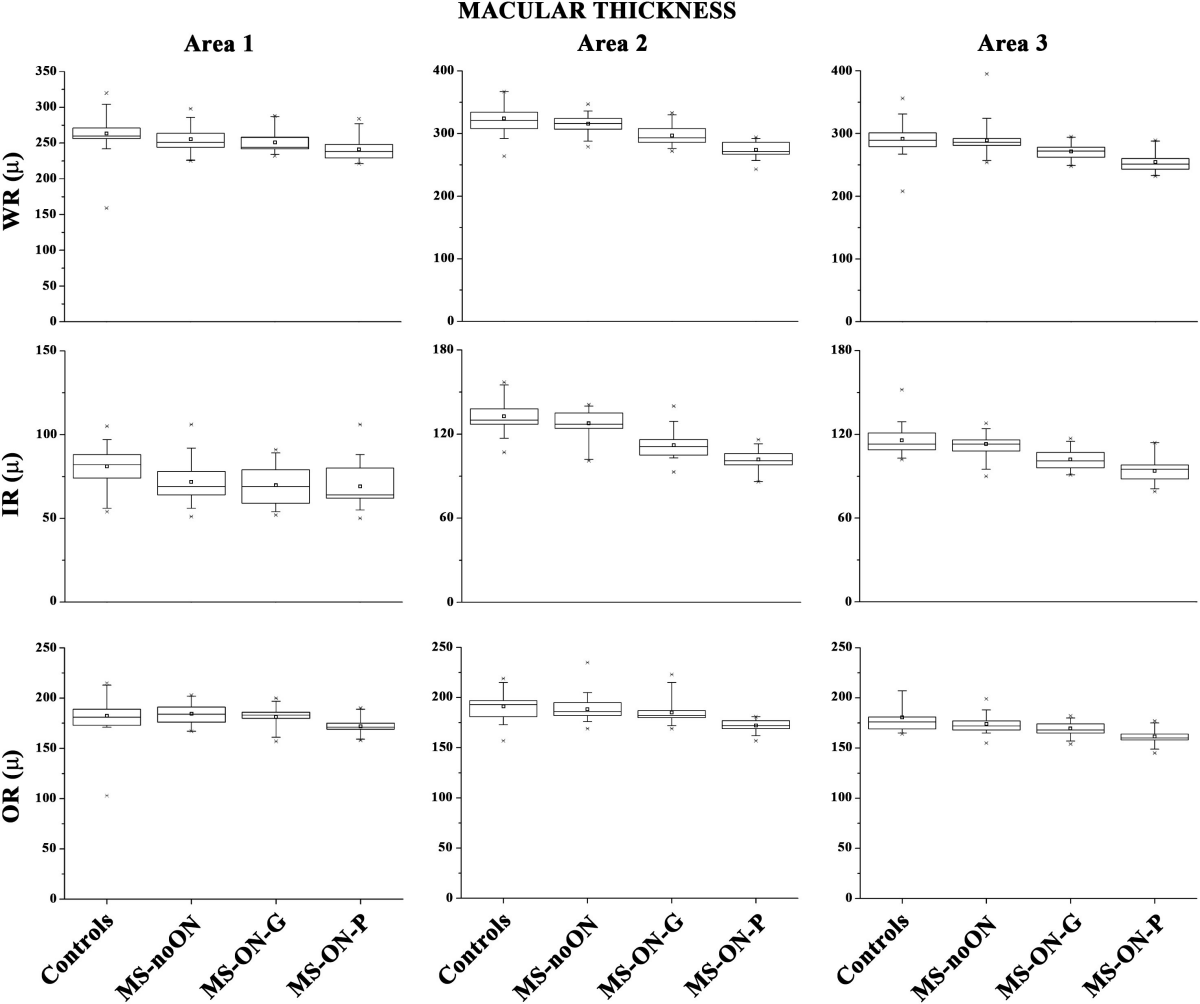
Box plots of Spectral domain-Optical Coherence Tomography macular thicknesses in control (C) eyes, Multiple Sclerosis patients without Optic Neuritis (MS-noON), with Optic Neuritis and good recovery of best corrected visual acuity (MS-ON-G), and with Optic Neuritis and poor recovery of best corrected visual acuity (MS-ON-P). WR-MT= Whole Retinal Macular Thickness; IR-MT =Inner Retinal Macular Thickness; OR-MT = Outer Retinal Macular Thickness; μ = micron; Area 1= circular area 1 mm centered to the fovea; Area 2 = annular area 1–3 mm centered to the fovea; Area 3 = annular area 3–6 mm centered to the fovea.

On Figure 2 we presented the box plots of the values of WR, IR, and OR MT measured from each localized area observed in the control, MS-noON, MS-ON-G, and MS-ON-P groups. On Table 3, we reported the statistical analysis between the groups.

**Table 3:** Spectral domain-Optical Coherence Tomography macular thickness (MT) segmentation analysis.

		**WR-MT**			**IR-MT**			**OR-MT**		
		**AREA 1**	**AREA 2**	**AREA 3**	**AREA 1**	**AREA 2**	**AREA 3**	**AREA 1**	**AREA 2**	**AREA 3**
**MS-noON** **vs. C**	*f* (1,74)	8.019	6.803	7.389	9.499	21.388	35.579	0.262	0.753	2.331
	*P*	** <0.01**	** <0.01**	** <0.01**	** <0.01**	** <0.01**	** <0.01**	0.609	0.391	0.131
**MS-ON-G** **vs. C**	*f* (1,61)	22.861	52.802	61.022	11.441	425.062	59.471	0.453	0.159	0.019
	*P*	** <0.01**	** <0.01**	** <0.01**	** <0.01**	** <0.01**	** <0.01**	0.503	0.692	0.977
**MS-ON-G** **vs. MS-noON**	*f* (1,66)	1.362	27.756	15.932	0.509	239.188	3.387	1.392	1.402	2.352
	*P*	0.248	** <0.01**	** <0.01**	0.478	** <0.01**	0.070	0.243	0.241	0.130
**MS-ON-P** **vs. C**	*f* (1,60)	53.75	228.65	139.13	12.20	287.14	114.34	23.42	35.14	120.61
	*P*	** <0.01**	** <0.01**	** <0.01**	** <0.01**	** <0.01**	** <0.01**	** <0.01**	** <0.01**	** <0.01**
**MS-ON-P** **vs. MS-noON**	*f* (1,65)	32.53	167.69	50.85	0.85	127.97	30.93	30.50	45.23	132.32
	*P*	** <0.01**	** <0.01**	** <0.01**	0.350	** <0.01**	** <0.01**	** <0.01**	** <0.01**	** <0.01**
**MS-ON-P** **vs. MS-ON-G**	*f* (1,52)	7.62	34.39	19.45	0.04	18.45	13.47	15.25	25.57	15.26
	*P*	** <0.01**	** <0.01**	** <0.01**	0.853	** <0.01**	** <0.01**	** <0.01**	** <0.01**	** <0.01**

*Results of statistical analysis (one-way analysis of variance) between groups: control (C) eyes, Multiple Sclerosis patients without Optic Neuritis (MS-noON), with Optic Neuritis and good recovery of best corrected visual acuity (MS-ON-G), and with Optic Neuritis and poor recovery of best corrected visual acuity (MS-ON-P). WR-MT, Whole Retinal Macular Thickness; IR-MT, Inner Retinal Macular Thickness; OR-MT, Outer Retinal Macular Thickness; Area 1 = circular area 1 mm centered to the fovea; Area 2 = annular area 1–3 mm centered to the fovea; Area 3 = annular area 3–6 mm centered to the fovea; p < 0.01 were considered as statistically significant for group comparisons and are expressed in bold*.

On average, in the MS-noON, MS-ON-G, and MS-ON-P groups the values of WR and IR MT detected in the Areas 1, 2, and 3, were significantly (*p* < 0.01) reduced with respect to the controls. With respect to the MS-noON group, in the MS-ON-G group a significant (*p* < 0.01) reduction of WR MT values were detected in the Areas 2 and 3, whereas IR MT values were significantly reduced (*p* < 0.01) exclusively in Area 2. The values observed in the MS-ON-P group were further significantly (*p* < 0.01) reduced with respect to those of the MS-noON and MS-ON-G groups, but the IR MT in Area 1.

When considering the OR MT from all Areas (1, 2, and 3), not statistically significant (*p* > 0.01) differences between the values observed in the controls, MS-noON, and MS-ON-G groups were found. On the contrary, the values of OR MT detected in all Areas (1, 2, and 3) in the MS-ON-P group were significantly (*p* < 0.01) reduced with respect to those of the controls, MS-noON, and MS-ON-G groups.

### Multiple Regressions Between Best Corrected Visual Acuity and Sd-OCT Macular Volume and Thickness Data

On Table 4, the results of multiple regressions between the individual values of WR, IR, and OR MV and MT and those of BCVA observed in the MS-ON-P group are shown. WR and IR volumes and thickness were significantly (*p* < 0.05) related with BCVA. Not significant (*p* > 0.05) relationships between OR volumes and thickness and BCVA were found.

**Table 4:** Multiple regression analysis between best corrected visual acuity (BCVA) and Spectral domain-Optical Coherence Tomography macular volume (A) and macular thickness (B) values in Multiple Sclerosis patients with Optic Neuritis and poor recovery of BCVA (MS-ON-P group).

	**Regression (F; p; *R*^**2**^)**	**AREA 1** **(F; p)**	**AREA 2** **(F; p)**	**AREA 3** **(F; p)**	**Regression equation**
**A**					
**WR-MV**	5.55; 0.005; 43.06%	4.89; 0.038	13.21; 0.001	5.44; 0.029	BCVA = 1.69 + 8.52A1–3.381A2 + 0.902A3
**IR-MV**	7.43; 0.001; 50.31%	4.68; 0.042	15.86; 0.001	5.18; 0.033	BCVA = 1.792 + 10.00A1–5.47A2 + 1.30A3
**OR-MV**	0.29; 0.833; 3.79%	0.00; 0.966	0.73; 0.401	0.18; 0.672	BCVA = −0.47–0.5A1 + 0.281A2 + 0.304A3
**B**					
**WR-MT**	5.74; 0.005; 43.89%	4.85; 0.038	13.65; 0.001	5.69; 0.026	BCVA = 1.77 + 0.007A1–0.022A2 + 0.012A3
**IR-MT**	7.25; 0.001; 49.71%	4.40; 0.048	15.46; 0.001	4.95; 0.037	BCVA = 1.800 + 0.008A1–0.034t2 + 0.016A3
**OR-MT**	2.16; 0.122; 22.72%	1.24; 0.278	5.21; 0.032	5.44; 0.029	BCVA = 0.27 + 0.010A1–0.032A2 + 0.024A3

*WR-MV, Whole Retinal Macular Volume; IR-MV, Inner Retina Macular Volume; OR-MV, Outer Retina Macular Volume; WR-MT, Whole Retinal Macular Thickness; IR-MT, Inner Retinal Macular Thickness; OR-MT, Outer Retinal Macular Thickness; Area 1= circular area 1 mm centered to the fovea; Area 2= annular area 1–3 mm centered to the fovea; Area 3 = annular area 3–6 mm centered to the fovea. The second column shows the regression model results and the third column shows F, p-values and coefficients' results*.

## Discussion

The purpose of this study was to investigate the morphology of the macular IR and OR layers in MS patients with and without a history of ON, followed by good or poor recovery of BCVA, to contribute to the controversial topic on the potential OR involvement in this neurodegenerative disorder.

The main results of the present study were that in MS patients without ON (the MS-noON group) and in MS patients with ON and a good recovery of BCVA (the MS-ON-G group) there was a morphological impairment of the IR layers, without changes of OR layers; in MS patients with ON and poor recovery of BCVA (the MS-ON-P group) there was a morphological impairment of both IR and OR layers.

All our results apply to a highly homogenous group of RR MS patients with not significant differences in age, MS-DD, and EDDS score.

In our work, the macular morphology was evaluated by the Sd-OCT assessment of segmented MV and MT. More commonly, automatic or manual segmentation of retinal layers' thicknesses, not volumes, is performed. This needs to be considered since different results obtained by using these morphological measurements could be a source of bias when comparing OCT studies.

Moreover, the literature has described reduced segmented OR (6, 15), and mainly IR layers (14, 17) MT, without the evaluation of MV, in MS eyes, mixing together eyes with and without ON in primary progressive and RR patients (6, 8, 12, 15).

For this reason and to add clarity on this matter, we present the discussion of MV and MT data separately in the three examined groups, as follows.

### Sd-OCT Data in Multiple Sclerosis Patients Without History of Optic Neuritis (MS-noON Group)

When comparing data with controls, in the MS-noON group we found similar values of WR, IR, and OR MV, but significantly reduced WR and IR MT values in all examined areas. The different results obtained by measuring volumes or thickness may be ascribed to the possibility that the retinal volume encloses not exclusively neuronal cellular elements, and specifically their soma, but also a large quantity of non-neuronal elements (such as astrocytes or Muller cells). Therefore, the finding that WR and IR MT were significantly reduced in this group, but not the MV, may be accounted to the specific impairment of all the neuro-retinal elements constituting the IR. Structural or non-neural elements are probably not involved in the neurodegenerative process of MS without ON.

The main result, however, relies on the finding that OR MV and MT were not significantly different with respect to controls, suggesting that the OR is spared from neurodegeneration even when no ON event occurs (12, 14, 16, 19). We are aware of different results in MS-noON patients that led Saidha et al. (15) to hypothesize the concept of “primary retinal pathology,” based on the finding of IR and OR thinning mainly in patients with progressive MS-noON. However, we studied more selectively RR MS patients and based on our results we cannot drive similar conclusive findings.

### Sd-OCT Data in Multiple Sclerosis Patients With History of Optic Neuritis and Good Recovery of Visual Acuity (MS-ON-G Group)

Regarding our Sd-OCT volume findings, in the MS-ON-G group we detected a significant reduction of WR and IR MV in the parafoveal areas (Area 2 and Area 3), as well as in the wide 6 mm area, but in Area 1 which represents the fovea. This suggests that the morphological IR (and WR) impairment after ON, accordingly with previous observations (6, 8, 10, 12, 14–17, 24, 28, 29), occurs mainly in the parafovea with absence of morphological impairment of the fovea. This can be explained by considering macular anatomical characteristics. Indeed, a greater proportion of RGCs per unit volume is present in the parafovea, whereas in the fovea and in the periphery of the macula, RGCs and RNFL are less represented. Because the RGCs comprise about 35% of the total WR thickness of the macula (20, 28, 30), the main impact on IR and WR reduction may regard RGCs. Therefore, our results on volume analysis suggest that in MS, in the presence of ON and when a good recovery of BCVA was reached after ON, the neural elements of the fovea are likely morphologically spared from the retrograde degeneration.

Similarly to our findings, in previous OCT studies performed in MS-ON eyes, a significant reduction of IR volume was found in MS patients with poor (10) (as our MS-ON-P group) or good recovery (as our MS-ON-G group) of BCVA (18, 29) after ON, measuring the MV from the entire macular area (our Area 1+2+3). Therefore, no specific information about the foveal morphological integrity or abnormality were given, and it is conceivable that the observed reduction of the IR volume was influenced by a greater structural involvement of the parafoveal neural elements.

Also, when comparing data between the MS-ON-G and MS-noON groups, significant differences in WR and IR MV were found in all areas but in Area 1, thus confirming that in this neurodegenerative disorder the fovea (in terms of MV values) remains structurally spared, independently from the ON.

Similar results of WR and IR were obtained when segmenting MT, however the foveal measurements from Area 1 were significantly impaired compared to controls. This could be explained considering the above-mentioned possibility of MV to capture structural or non-neural elements which are not enclosed in the MT measurement.

By contrast, we found relative equivalent results in terms of MV and/or MT about OR values that were not significantly different from those of controls, suggesting that OR foveal and parafoveal elements are morphologically spared by the post-neuritis degenerative process; it is likely that this condition should induce the recovery of good high-contrast BCVA, as detected in our MS-ON-G cohort. This structural finding is in agreement with a previous report by Hanson et al. (29), who observed a not significant reduction of segmented OR volume in MS-ON patients with complete recovery of visual dysfunction who recovered a good high-contrast BCVA (< 0.2 logMAR), similarly to our enrolled MS-ON-G patients, and had no major visual field defects. Also other authors (16–18) identified the absence of thinning of the OR layers after ON in patients with recovery of high-contrast BCVA.

### Sd-OCT Data in Multiple Sclerosis Patients With History of Optic Neuritis and Poor Recovery of Visual Acuity (MS-ON-P Group)

In our cohort of MS-ON with poor recovery of BCVA, we found reduced MV and MT values of WR in all areas, as well as MT of IR, compared to controls. Only MV values of IR from Area 1 were not significantly reduced when compared to the controls, MS-noON, and MS-ON-G groups. This means that also in MS after an event of ON, when BCVA is not fully recovered, as expected, WR and IR layers from the parafoveal areas are structurally impaired.

In the same group, however, differently from the MS-noON and MS-ON-G groups, we found significant abnormal OR MVs and MTs from all examined areas, thus assessing the pre-ganglionic elements morphological impairment. Our morphological results could indicate that the post-neuritis retrograde degeneration might also involve the elements located in the OR, impacting the neuronal chain of the fovea. Therefore, the wider morphological concomitant involvement of OR and IR layers could explain the absence of good recovery of BCVA in MS-ON-P patients with respect to MS-ON-G patients, in which an exclusive IR structural impairment was detected. These findings were also supported by adaptive optics data in optic neuropathies, including MS ON, by Choi et al. (31) who described photoreceptor structural abnormalities, when there is permanent damage to overlying IR layers.

The OR morphological changes were not related to BCVA decrease (see Table 4). It seems that in the reduction of BCVA, the main contribution is given by the morphological changes of the parafoveal IR layers, as also described in previous studies (18, 19, 28, 32) where it was found that GCL+IPL thinning is most significantly correlated with reduced high contrast BCVA in MS-ON patients, similarly to our cohort, at least 6 months after the ON occurrence (24).

### Conclusive Remarks and Neurophysiological Hypotheses

We acknowledge that the Sd-OCT device used could not provide high definition segmentation data layer-by-layer of the OR and IR in both MS groups as a limitation of our study (33). However, by finding congruent results of OR integrity by using either volume and thickness segmentation analyses, we are confident that this limitation has been overcome.

In summary, our main findings led us to make some relevant conclusions and hypotheses: (1) in our selected cohort of RR MS patients, the well-known (6, 8, 10, 12, 14–17, 24, 28, 29) morphological involvement of the IR is confirmed with more exhaustive information provided by MT assessment rather than MV analysis, in specific localized areas; (2) no morphological abnormalities can be found at the level of the OR in absence of ON; by contrast, in occurrence of ON with good recovery of BCVA, it is likely that the OR layers are preserved from the extent of the neurodegenerative process, and, in the absence of exhaustive data in literature, it can be hypothesized that in this case the retinal elements located outside the OR (i.e., middle retina) could play a role to counteract neurodegeneration; (3) by contrast, when there is an absence or an inadequate previously supposed protective role, then the morphological impairment extends also to OR structures and this, together with the IR damage, leads to poor recovery of BCVA.

Our hypotheses that retinal synaptic elements located between OR and IR layers are relevant for neuroinflammatory changes in MS and that the homeostasis of the middle retina is crucial to counteract MS-related neurodegeneration can be supported by preclinical (34, 35) and clinical (14, 15) evidences.

In fact, an early synaptic pathology occurs in well-validated MS mouse models of ON, altered synaptic vesicle cycling in ribbon synapses of the myelin-free retina was reported, which are likely targeted by an auto-reactive immune system process (34). The auto-immune response in these animal models is directed against two adhesion proteins (CASPR1/CNTN1) (36), that are present both in the paranodal region of myelinated nerves as well as at retinal ribbon synapses (34). Related to this topic, the retina has been considered a primary immune target in MS and in MS-related optic neuritis in many previous clinical studies (14, 15).

In order to better understand the role of middle retinal elements in this process, further studies on both experimental and clinical sides are needed.

## Data Availability Statement

The raw data supporting the conclusions of this article will be made available by the authors, without undue reservation.

## Ethics Statement

The studies involving human participants were reviewed and approved by Comitato Etico Centrale IRCCS Lazio, Sezione IFO/Fondazione Bietti, Rome, Italy. The patients/participants provided their written informed consent to participate in this study.

## Author Contributions

LZ, VP, and DC: Concept and design. LBa, MA, and LBo: data collection. VP: statistical expertise. VP, LZ, and DC: analysis and interpretation. LZ, VP, and LBa: writing the article. VP, LZ, DC, and AG: critical revision of the article. VP, LZ, and DC: final approval of the article. All authors: reviewed the manuscript and agreed to be accountable for all aspects of the work.

## Conflict of Interest

The authors declare that the research was conducted in the absence of any commercial or financial relationships that could be construed as a potential conflict of interest.
